# Respiratory Support in Bronchiolitis: A Narrative Review of the Evidence for and Against High-Flow Nasal Cannula (HFNC) Use

**DOI:** 10.7759/cureus.101297

**Published:** 2026-01-11

**Authors:** Joseph O Odeyemi

**Affiliations:** 1 Internal Medicine/Pediatrics, University of Kansas School of Medicine-Wichita, Wichita, USA

**Keywords:** high-flow nasal canula, hypoxia, low-flow oxygen delivery, respiratory distress, viral bronchiolitis

## Abstract

Bronchiolitis is a leading cause of pediatric hospitalization, and the use of high-flow nasal cannula (HFNC) as respiratory support has increased substantially over the past decade despite ongoing uncertainty about its clinical benefits. This narrative review evaluates the evidence supporting and questioning HFNC use in infants and young children with bronchiolitis. Early observational studies suggested reductions in intubation rates and improved clinical parameters with HFNC; however, more recent randomized controlled trials show no consistent benefit in length of stay, duration of oxygen therapy, pediatric intensive care unit (PICU) transfer rates, or need for mechanical ventilation when compared with conventional low-flow oxygen. HFNC may reduce treatment failure among children who do not respond to low-flow therapy, but its expanding use has been associated with higher healthcare costs, increased PICU admissions, and potential complications such as aerophagia, delayed enteral feeding, and air leak syndromes. Evidence remains limited regarding HFNC use in non-hypoxic children with respiratory distress, and current guidelines do not support “oxygen for comfort.” A stepwise, evidence-guided approach to respiratory support, grounded in objective bronchiolitis scoring systems, may help prevent overuse and optimize outcomes. Further research is needed to clarify indications for HFNC, define standardized initiation and escalation criteria, and evaluate its role in non-hypoxic bronchiolitis.

## Introduction and background

Bronchiolitis is one of the most common infections in children under two years old. It typically begins with upper respiratory symptoms and progresses to fever, cough, and respiratory distress. Bronchiolitis is a viral illness, and the most implicated virus is the respiratory syncytial virus (RSV) [1]. The clinical syndrome of bronchiolitis can range from mild respiratory symptoms to severe respiratory failure requiring intensive care and invasive ventilation. Bronchiolitis is a leading cause of illness and hospitalization in infants and toddlers, with a reported yearly incidence of up to 20% [1,2]. Additionally, the management of bronchiolitis in children under two years costs the United States health care system billions of dollars annually [2]. While most children experience mild, self-limited disease, a subset develop hypoxia or respiratory failure requiring escalated levels of support. High-flow nasal cannula (HFNC) therapy has become increasingly utilized in the management of bronchiolitis, often initiated early in the hospital course. However, its rapid adoption has outpaced the evidence supporting its efficacy, leading to variability in practice and rising healthcare costs. Evaluating the benefits and limitations of HFNC is essential for guiding appropriate use and optimizing outcomes. This narrative review aims to evaluate the current evidence for and against the use of HFNC in children with bronchiolitis, highlighting its benefits, limitations, and areas requiring further research.

## Review

Pathophysiology of viral bronchiolitis 

The virus infects the epithelial cells of the small airways, causing inflammation [1]. This leads to cell death, sloughing of the epithelial tissue, airway edema, accumulation of exudates, and dysfunction of the respiratory cilia [1]. These changes result in partial obstruction of the airway, reduced lung compliance, and air trapping and can lead to atelectasis [1]. These pathophysiological changes manifest clinically as respiratory distress and hypoxia. Moreover, the obstructive pathophysiology of bronchiolitis is the reason these children often have expiratory wheezing [1].

Respiratory support in bronchiolitis

Children with bronchiolitis may experience respiratory distress and hypoxia, which can progress to respiratory failure in some cases. As a result, one of the key management strategies for viral bronchiolitis is providing respiratory support. The level of support can vary from a low-flow nasal cannula (LFNC) to invasive ventilation depending on the severity of the patient’s illness. In many institutions, respiratory support is escalated in a stepwise manner, moving from LFNC to high-flow nasal cannula (HFNC), then to non-invasive ventilation (NIV), and ultimately to endotracheal intubation for mechanical ventilation if needed. The decision to escalate respiratory support varies between clinicians and institutions and is usually based on a combination of the patient's clinical presentation, oxygen saturation (SpO2), and other indicators of oxygenation and ventilation [1-3].

There are specific indications for initiating oxygen supplementation in children with bronchiolitis. According to the 2021 National Institute for Health and Care Excellence (NICE) guidelines, oxygen should be administered to children with bronchiolitis who are older than six weeks and have a SpO2 persistently below 90% or to children under six weeks or those with a chronic cardiopulmonary condition if their SpO2 is less than 92% [4]. Additionally, the American Academy of Pediatrics (AAP) recommends that providers may opt not to administer oxygen supplementation in bronchiolitis patients with an SpO2 above 90% [3]. This threshold is based on the understanding that an oxyhemoglobin saturation greater than 89% is sufficient for adequate tissue oxygenation [3]. The guidelines do not recommend starting supplemental oxygen or escalating respiratory support based solely on the presence of respiratory distress, though it may be indicated in cases of worsening distress or impending respiratory failure [5]. Of note, previous European guidelines had recommended supplemental oxygen for children with respiratory distress and SpO2 levels between 90% and 94% [5]. However, a recent systematic review found that starting oxygen supplementation at these thresholds may lead to higher hospitalization rates and longer hospital stays [6].

High-flow nasal cannula use in bronchiolitis

HFNC was introduced into clinical practice around 20 years ago as an alternative to continuous positive airway pressure (CPAP) for managing apnea of prematurity [7]. Over the past decade, HFNC has been increasingly used for children with bronchiolitis who experience significant respiratory distress, raising questions about its benefits and potential downsides [8]. In a large cohort study involving over two million children in Canada, Mahant et al. found that bronchiolitis accounted for approximately 13.3% of all hospitalizations in children under two years between 2004 and 2018 [8]. During this period, the hospitalization rates and mortality rates for bronchiolitis remained stable; however, the rates of HFNC use, intensive care unit (ICU) admission, and hospitalization costs went up significantly [8]. This suggests that the increased use of HFNC may have contributed to higher ICU admission rates, without a corresponding reduction in patient mortality [8].

High-flow nasal cannula (HFNC) therapy offers several physiologic benefits that support respiratory function. By delivering flows that meet or exceed a patient’s inspiratory demand, HFNC can help overcome airway resistance [7,9]. The system also provides heated, humidified air, which reduces the patient’s energy expenditure and improves mucociliary function by maintaining airway hydration [7,9]. In addition, HFNC delivers a fixed fraction of inspired oxygen (FiO₂) to the alveoli by minimizing ambient air mixing [7,9]. HFNC may also increase functional residual capacity (FRC), helping keep alveoli open during expiration and reducing the risk of atelectasis, likely due to the small degree of positive airway pressure generated, an effect that is particularly notable in infants [7,9]. Finally, HFNC promotes nasopharyngeal dead-space washout, thereby reducing rebreathing and supporting more efficient ventilation [7,9].

Initial evidence supporting the use of HHFNC

A retrospective chart review compared infants under two years old admitted for bronchiolitis the year before high-flow nasal cannula (HFNC) was introduced at a children's hospital in Massachusetts with those admitted during the first year after HFNC was introduced in 2006 (Table 1). The study aimed to determine whether HFNC reduced intubation rates for infants with bronchiolitis. The results showed a statistically significant decrease in both intubation rates and PICU length of stay following the introduction of HFNC [10].

**Table 1:** Summary of results from McKiernan et al. comparing the type of respiratory support used for bronchiolitis and other relevant parameters in the year prior to the introduction of HFNC to the first year after the introduction of HFNC. LFNC: Low-flow nasal cannula, HFNC: High-flow nasal cannula, CPAP: Continuous positive airway pressure, PICU: Pediatric intensive care unit

Respiratory support	Year before HFNC 05/06 (n=57)	First year of HFNC 06/07 (n=58)
Room air	9 (15.8%)	1 (1.7%)
Blow-by oxygen	9 (15.8%)	1 (1.7%)
LFNC	33 (57.9%)	5 (8.6%)
Simple mask	1 (1.8%)	0
Non-rebreather face mask	2 (3.5%)	0
CPAP	3 (5.3%)	0
HFNC	0	51 (87.9%)
Intubation with mechanical ventilation	13 (23%)	5 (9%)
Median PICU length of stay	6 days	4 days

Another retrospective chart review conducted in 2011 examined infants under two years old with bronchiolitis who were admitted to the PICU for increased respiratory support over a five-year period from 2005 to 2009, following the introduction of HFNC at this hospital [11]. This study explored the impact of HFNC on the need for other noninvasive or invasive respiratory supports. It reported a significant reduction in intubation rates among PICU patients with bronchiolitis, dropping from 37% in 2005 to 7% in 2009 [11]. By the end of the five-year period, two-thirds of these patients were treated with HFNC, compared to just 13% in 2005 [11]. However, despite the reduction in intubation rates, the study found no difference in the length of hospital stay [11].

Furthermore, a 2016 literature review examining the evidence for HFNC use in bronchiolitis identified 13 relevant studies. Among these, 10 were observational studies, one was a case-control study, and two were non-blinded randomized controlled trials comparing HFNC to hypertonic saline and HFNC to head-box oxygen [12]. Pertinently, four of these studies compared HFNC to other forms of supplemental oxygen therapy for managing bronchiolitis (Table 2) [12].

**Table 2:** Summary of the key findings in the reviewed studies from Mikalsen et al. HFNC: High-flow nasal cannula, LFNC: Low-flow nasal cannula, PICU: Pediatric intensive care unit

Author (year)	Study design	Participants	Key results
Hillard, 2012 [13]	Prospective interventional randomized, unblinded	Nineteen infants under 12 months with bronchiolitis were randomized to receive either head-box oxygen (n = 8) or HFNC (n = 11).	Median peripheral capillary oxygen saturation (SpO2) was higher in the HFNC group at 8hr and 12hr but similar at 24hr. Fraction of inspired oxygen (FiO2) was higher in the HFNC group at all 3 time points.
Mayfield, 2014 [14]	Observational case control	61 infants under 12 months with bronchiolitis treated with HFNC. 33 infants treated with LFNC.	The risk of PICU admission was four times higher in the LFNC group compared to the HFNC group.
Milani, 2016 [15]	Prospective observational	36 children under 12 months hospitalized with bronchiolitis.18 infants treated with HFNC, 18 with LFNC.	Improvements in respiratory rate, respiratory effort and ability to feed faster in HFNC group. HFNC group needed oxygen for 2 days less and length of stay was 3 days shorter than the LFNC group.
Metge, 2014 [16]	Retrospective observational	34 children under 12 months admitted to the PICU with bronchiolitis. 19 children treated with continous positive airway pressure (CPAP); 15 children treated with HFNC.	No difference between the groups in length of stay, respiratory rate, partial pressure of carbon dioxide in arterial blood (PaCO2), FiO2 and duration of oxygen support.

The Problem With This Evidence

Most of these studies were retrospective and subject to various biases and subjectivity. Additionally, many were conducted at single institutions, which may limit their generalizability.

Current evidence

A 2020 systematic review examined relevant randomized controlled trials on the efficacy and safety of HFNC conducted between 2000 and 2020 [17]. The review's inclusion criteria required that studies be randomized controlled trials involving only children under two years old with acute bronchiolitis, focus on the efficacy and/or safety of HFNC, and be published in English [17]. The review included seven studies: four compared conventional supplemental oxygen therapy to HFNC, and three compared HFNC to CPAP (Table 3) [17].

**Table 3:** Summary of the results from trials comparing HFNC to supplemental oxygen therapy (SOT) in the treatment of bronchiolitis, as reviewed by Moreel & Proesmans, 2020. LOS: Length of stay in hospital, LFNC: Low-flow nasal cannula, HFNC: High-flow nasal cannula, PICU: Pediatric intensive care unit, FiO2: Fraction of inspired oxygen, N/A: Not applicable Treatment failure was defined as meeting three out of the following four criteria: unchanged or increasing heart rate or respiratory rate, a higher oxygen requirement than before, and an elevated hospital early warning tool or respiratory distress score [17]. Clinicians also had the discretion to escalate therapy based on additional clinical concerns not accounted for in the four specified criteria [17].

Author (year)	Sample size (HFNC/ Control)	Age	Oxygen therapy (HFNC/Control)	Treatment failure (HFNC vs. Control)	PICU transfer (HFNC vs. Control)	LOS (days) (HFNC vs. Control)	Duration of oxygen therapy (HFNC vs. Control)
Ergul (2018) [18]	30/30	<2 yrs	1L/kg/min, Max 20L| 10-15L/min with oxymask	0/30 (0%) vs. 7/30 (23%) (p = 0.009)	N/A	4 days vs. 5 days (p < 0.001)	2.3 days vs. 4.0 days (p < 0.001)
Franklin (2018) [5]	739/733	<1 yr	2L/kg/min FiO2 40%| 100% FiO2 max 2L/min using LFNC	87/739 (12%) vs. 23% 167/733 (23%) (p < 0.001)	12% (87/739) vs. 9% (65/733) (p = 0.08)	3.1 days vs. 2.9 days (p = 0.19)	1.8 days vs. 1.9 days (p = 0.61)
Kepreotes (2017) [19]	101/101	<2 yrs	1L/kg/min, Max 20L FiO2 60%| 100% FiO2 max 2L/min using LFNC	14/101 (14%) vs. 33/101 (33%) (p = 0.0016)	14% (14/101) vs. 12% (12/101) (p = 0.41)	2.0 days vs. 2.0 days (p = 0.99)	0.8 days vs. 1.0 days (p = 0.61)
Hilliard (2012) [13]	8/11	<1 yr	4-8 L/min| Supplemental oxygen therapy through head-box	No treatment failure	No PICU transfers	6.7 days vs. 6.8 days (p = 0.7)	4.9 days vs. 3.3 days (p = 0.32)

These RCTs found no significant difference in PICU transfer rates or intubation rates between patients treated with conventional supplemental oxygen therapy (SOT) and those treated with high-flow nasal cannula (HFNC) [17]. Similarly, there were no differences in the length of stay or duration of oxygen therapy in three out of the four RCTs, including the two studies with large sample sizes [17]. However, treatment failure rates were higher in patients receiving conventional SOT compared to those on HFNC [17]. Notably, about 60% of the children who experienced treatment failure on conventional SOT were successfully switched to HFNC without an increased risk of PICU transfer or further escalation of respiratory support [17].

More recently, a 2024 meta-analysis of 11 RCTs and quasi-RCTs, involving over 2,000 infants under 24 months with clinical bronchiolitis, compared HFNC to low-flow oxygen therapy [20]. The analysis found a significant reduction in the total duration of hospital stay for the HFNC group, with a mean difference of 0.65 days (p < 0.00001) [20]. It also reported a decrease in the duration of oxygen therapy by 0.59 days (p < 0.00001) and a reduced risk of escalation in respiratory support, with a risk ratio of 0.55 (95% CI 0.39 to 0.79, p = 0.001) for the HFNC group [20].

Another recently published multicenter study, the PARIS 2 randomized clinical trial, compared early high-flow oxygen therapy to standard oxygen therapy for acute hypoxic respiratory failure in children aged 1 to 4 years [21]. The study included a sample size of 1,567 children, who were randomized in a 1:1 ratio to the two groups [21]. Unlike similar studies focused solely on bronchiolitis, this trial included children within the specified age group who met the following criteria: increased work of breathing, oxygen requirement to maintain a prespecified oxygen saturation (92% in most hospitals, except one that used 90%), tachypnea with a respiratory rate greater than 35 breaths per minute, and need for hospitalization [21].

The primary outcome was the length of hospital stay [21]. The study found a statistically significant increase in length of stay in the high-flow oxygen group compared to the standard oxygen therapy group (1.77 days vs. 1.5 days, p < 0.001) [21]. This difference remained significant when participants were stratified into groups with wheezing (obstructive airway disease) and without wheezing (non-obstructive airway disease). The high-flow oxygen group also required a longer duration of oxygen therapy [21]. Additionally, ICU admissions were significantly higher in the high-flow oxygen group compared to the standard oxygen therapy group (12.5% vs. 6.9%) [21].

HFNC use in non-hypoxic patients

Many interventions in bronchiolitis remain poorly studied, including the use of high-flow oxygen therapy at 21% FiO2, in non-hypoxic bronchiolitis patients with respiratory distress. High-flow oxygen therapy can influence respiratory mechanics and physiological parameters in ways that may theoretically improve work of breathing even in non-hypoxic patients [7,9].

The Milani et al. study conducted in 2016 compared high-flow nasal cannula (HFNC) to low-flow oxygen delivery in children with moderate to severe bronchiolitis [15]. It assessed respiratory rate, breathing work, and feeding ability. The study found a statistically significant reduction in respiratory rate in the HFNC group, evident as early as 30 minutes after initiation [15]. Additionally, improvements in respiratory effort and feeding ability were statistically significant in the HFNC group compared to the low-flow oxygen delivery group [15]. Notably, oxygen supplementation in this study was initiated at SpO₂ levels below 92% and titrated to achieve SpO₂ levels of 94% or higher [15].

Other studies have reported similar improvements in breathing patterns and respiratory distress with HFNC, with various mechanisms proposed [22,23]. However, most studies focus on children with hypoxia [22,23]. Further research is needed to evaluate HFNC specifically for non-hypoxic children with respiratory distress. Furthermore, the concept of "oxygen for comfort," involving oxygen support for respiratory distress without hypoxia, lacks strong evidence [24]. A 2021 cross-sectional survey of pediatric nurses, respiratory therapists (RTs), and physicians found that a significant proportion of providers believed oxygen support could improve work of breathing in non-hypoxic children [24]. Specifically, half of the nurses, one-third of the RTs, and a quarter of the other providers endorsed this practice. However, this practice is not formally defined and is not recommended by any guidelines [24].

Discussion

Available data suggest that high-flow nasal cannula (HFNC) can be effective for children who have not responded to low-flow nasal cannula (LFNC) and other conventional oxygen delivery methods. However, the data is variable and not very consistent. It is also important to note that HFNC use instead of LFNC may not reduce length of stay, duration of oxygen therapy, or intubation rates in patients with bronchiolitis. Additionally, HFNC can increase healthcare costs and ICU admissions. There are also specific drawbacks to HFNC use, such as aerophagia, which can cause abdominal discomfort and potentially interfere with enteral nutrition, while also increasing the risk of aspiration [9]. Some institutions even consider HFNC at certain flow rates a contraindication to feeding, which is particularly significant for infants and toddlers with limited nutrient reserves [9]. Delays in establishing enteral feeding may extend hospitalization. Furthermore, children on HFNC face a higher risk of complications, including absorption atelectasis and air leak syndromes such as pneumothorax and pneumomediastinum, which are generally more common at higher flow rates [25].

Given the available evidence and the associated risks and benefits, a stepwise approach to respiratory support in children with bronchiolitis, starting with low-flow nasal cannula (LFNC) and escalating to high-flow nasal cannula (HFNC) based on objective scoring systems rather than subjective signs of respiratory distress alone, would be prudent. Many institutions already utilize bronchiolitis scoring systems and protocols for escalating support. However, decisions to escalate are often influenced by subjective judgments due to the perception that HFNC is superior to LFNC for respiratory distress [26]. It is crucial for institutions to ensure that staff are informed about the evidence regarding HFNC use, its potential drawbacks, and the benefits of adopting a more conservative approach when appropriate [26].

Some current interventional strategies successfully employed by various institutions to reduce HFNC overuse in children with bronchiolitis include developing HFNC initiation criteria, providing staff education through the creation and distribution of bronchiolitis toolkits containing standardized protocols, and implementing team huddles to assess the ongoing need for HFNC in patients already admitted on this therapy [27].

While strict guidelines for HFNC use in bronchiolitis are still lacking, a 2025 specialist panel developed recommendations based on available evidence and expert consensus [28]. The panel advised that HFNC should be initiated only for respiratory distress and hypoxemia refractory to LFNC or in cases of impending respiratory failure, and that starting HFNC solely based on a patient’s respiratory score may be inappropriate [28]. They also highlighted limitations in both the quality of the evidence and the composition of the panel, emphasizing the need for continued research given the high prevalence of bronchiolitis and widespread HFNC use in pediatric hospitals [28].

Current gaps in the literature include the use of HFNC with 21% FiO2 solely for managing respiratory distress in non-hypoxic patients with bronchiolitis. This is significant because an argument can be made that using HFNC at room air (21%) constitutes respiratory support rather than oxygen supplementation and may benefit children with substantial work of breathing who are adequately oxygenating. Additionally, the development of standardized criteria for initiating and escalating HFNC in viral bronchiolitis by relevant professional bodies would be valuable. Furthermore, strategies to balance the clinical benefits of HFNC with its financial and logistical costs warrant further exploration.

## Conclusions

High-flow nasal cannula (HFNC) is a valuable tool in the management of bronchiolitis, particularly for children who fail to respond to low-flow oxygen therapies or children with severe diseases. While it offers significant benefits in improving respiratory distress and reducing treatment failure rates, its use should be balanced against potential drawbacks, such as increased ICU admissions, healthcare costs, and feeding-related challenges. A stepwise approach to initiating or escalating respiratory support, guided by objective scoring systems, can help optimize outcomes while minimizing adverse effects. Further research is warranted to refine the indications for HFNC and better understand its role in non-hypoxic patients with respiratory distress.
